# Defining cognitive profiles of depressive patients using the Brief Assessment of Cognition in Affective Disorders

**DOI:** 10.7717/peerj.7432

**Published:** 2019-08-01

**Authors:** Ruei-An Chen, Chun-Yi Lee, Yu Lee, Chi-Fa Hung, Yu-Chi Huang, Pao-Yen Lin, Sheng-Yu Lee, Liang-Jen Wang

**Affiliations:** 1 Department of Psychiatry, Kaohsiung Chang Gung Memorial Hospital and Chang Gung University College of Medicine, Kaohsiung, Taiwan; 2 Department of Psychiatry, Kaohsiung Veterans General Hospital, Kaohsiung, Taiwan; 3 Department of Psychiatry, College of Medicine, National Yang-Ming University, Taipei, Taiwan; 4 Department of Child and Adolescent Psychiatry, Kaohsiung Chang Gung Memorial Hospital and Chang Gung University College of Medicine, Kaohsiung, Taiwan

**Keywords:** Neurocognitive tests, Emotion, Depression, Attention, BACA

## Abstract

**Background:**

Cognitive impairments in patients with depressive disorders have a negative impact on their daily skill functioning and quality of life. In this study, we evaluated the cognitive profiles and associated factors of patients with depressive disorders with the Brief Assessment of Cognition in Affective Disorders (BAC-A).

**Methods:**

This cross-sectional study consisted of 75 patients with depressive disorders (56 patients with major depressive disorder (MDD) and 19 patients with depressive disorder NOS or dysthymic disorder (non-MDD)). We evaluated the participants’ cognitive functions at euthymic status using the BAC-A. The BAC-A includes six subtests derived from the Brief Assessment of Cognition in Schizophrenia (BAC-S) and Affective Processing Tests. The current severity of depressive symptoms was assessed with the 17-item Hamilton Depression Rating Scale (HAMD-17), and we recorded any psychotropic drugs being used by the patients.

**Results:**

We observed no differences in cognitive profiles in the MDD group and non-MDD group after adjusting for educational levels, severity of depression, and psychotropic drugs. Instead, the HAMD-17 scores were negatively correlated to cognitive performance in working memory, motor speed, verbal fluency, attention and processing speed, executive function, composite score, and the six indexes of the Affective Processing Test measured by the BAC-A. A longer illness duration was associated with worse performance of four indexes of the Affective Processing Test. Furthermore, benzodiazepine use was associated with a worse performance of verbal memory, and antidepressant use was associated with better motor speed performance.

**Conclusion:**

The current severity of depressive symptoms and psychotropic drugs being taken, not the diagnosis category, are associated with cognitive impairments in patients with depressive disorders. Clinicians should pay particular attention to managing residual depressive symptoms and prescribing adequate psychotropic drugs in order to eliminate depressive patients’ cognitive deficits.

## Introduction

Depressive disorder, including major depressive disorder (MDD), depressive disorder NOS, or dysthymic disorder, is among the leading causes of burden within the general population (Ferrari et al., 2013). Impaired cognitive function has been widely reported in patients with depressive disorder  (Burt, Zembar & Niederehe, 1995) and affects nearly two-thirds of depressed patients (Afridi et al., 2011; Butters et al., 2004), which subsequently causes significant disabilities in people’s lives. Previous evidence has indicated that reduced cognition in patients with MDD is closely related to an impaired quality of life and diminished personal function, while also contributing to disability (Jaeger et al., 2006; Naismith et al., 2007).Therefore, an adequate assessment for cognition decline in patients with depressive disorders has become a matter of great concern.

Cognitive impairment in such domains as complex attention, executive function, learning and memory, psychomotor skills, and processing speed has been proved in both acute episodes of depressive disorder and the euthymic mood state (Marazziti et al., 2010; Lee et al., 2012; Bora et al., 2013; Bortolato, Carvalho & McIntyre, 2014). However, the relationship between medication and cognitive function in patients with MDD and dysthymic patients is still poorly understood, and whether cognitive function may improve after treatment remains unknown.

Cognitive function in patients with MDD is usually assessed by using a well-developed cognitive assessment tool, such as the Screen for Cognitive Impairment in Psychiatry or the Cambridge Neuropsychological Test Automated Battery . Compared to the aforementioned batteries, the Brief Assessment of Cognition in Affective Disorders (BAC-A) was developed to evaluate cognitive deficits specifically in patients with affective disorder (Bauer et al., 2015). BAC-A includes six subtests derived from the Brief Assessment of Cognition in Schizophrenia (BAC-S), including the Controlled Oral Word Association, Tower of London, Token Motor Task, Digit Sequencing Task, Category Instances (Animals), Symbol Coding, and List Learning tests (Keefe et al., 2014). Such Affective Processing Tests as the Emotional Inhibition Test, Delayed Recognition Test, and Affective Interference Test were also included in the BAC-A (Bauer et al., 2015). BAC-A has been proven to be a reliable battery compared to traditional neuropsychological exams (Keefe et al., 2014) and has been validated for identifying cognitive impairment in patients with bipolar disorder (Bauer et al., 2015). However, the BACA has not yet been used in MDD for further profiling, and whether BACA is able to detect cognitive deficits in depressive patients even with residual symptoms remains unclear.

### Aims of the study

Previous studies have demonstrated the potential benefit of differentiating patients with bipolar disorder from those with unipolar depression at euthymic status through BAC-A (Lee et al., 2018b). Nevertheless, the effects of the different characteristics of depressive disorder and psychotropic medications on the performance of BAC-A have not yet been clearly identified. We proposed that patients with MDD diagnosis, those with residual depressive symptoms had poorer BACA performance than their counterparts. Therefore, this study aimed to investigate the cognitive profiles of patients with depressive disorders by using the BAC-A for neuropsychological assessment and to explore the effects of depression characteristics on the performance of each BAC-A index.

## Material and Methods

### Study participants

This cross-sectional study at Chang Gung Memorial Hospital was approved by the hospital’s Institutional Review Board (IRB No: 104-7324B). We carried out the procedures of this study in accordance with the Helsinki declaration and the ethical standards of the institutional and/or national research committee. Informed consent was obtained from all the participants in this study. All participants were paid 250 New Taiwan Dollars (approximately eight US dollars) for participating in this study.

Patients with a depressive disorder were recruited from the outpatient department of Kaohsiung Chang Gung Memorial Hospital and Kaohsiung Veterans General Hospital. The eligibility criteria for patients included the following: (a) diagnosis of a depressive disorder according to the criteria of the Diagnostic and Statistical Manual of Mental Disorders, Fourth Edition (DSM-IV-TR); (b) no known neurological or systemic diseases affecting cognitive performance; (c) age ≥ 18 years; and (d) able to speak and read Mandarin Chinese and provide informed consent. We recruited 75 patients with major depressive disorder (MDD), depressive disorder NOS or dysthymic disorder and interviewed them, while neuropsychological tests were performed in a euthymic state.

We previously recruited 220 healthy subjects to develop the norms of the Chinese BAC-A, and the finding had been published elsewhere (Lee et al., 2018a). In brief, the healthy subjects consisted of healthy individuals recruited from the employees of Kaohsiung Chang Gung Memorial Hospital, Kaohsiung Veterans General Hospital, and Keelung Chang Gung Memorial Hospital, as well as from community volunteers in Kaohsiung City and Keelung City, Taiwan. We herein selected 75 healthy subjects who were age- and sex-matched to our depression patients for making neurocognitive comparisons.

### Cognitive assessment

The Brief Assessment of Cognition in Schizophrenia (BACS) was administered to evaluate the cognitive functions of all participants  (Keefe et al., 2004). The BACS consists of tests with high test–retest reliability for measuring the cognitive deficits in schizophrenic patients (Keefe et al., 2006). The BAC-A contains all six subtests of the BACS and adds the Affective Processing Test. The Chinese version of the BAC-A has also been adapted and established by the original authors. Our research team established the normative data of the Chinese version of the BACS (Wang et al., 2017), which provides satisfactory psychometric properties (Wang et al., 2016). Furthermore, a previous study suggested that the Chinese BAC-A may contribute to differentiating unipolar depressive disorder from bipolar disorder patients in clinical settings (Lee et al., 2018b).

#### BACS subtests

Traditional neurocognitive domains such as working memory, motor speed, verbal fluency, attention and processing speed, verbal memory, and executive function were assessed using the following six subtests: the Digit Sequencing Task, List Learning Test, Token Motor Task, Controlled Oral Word Association Test, Category Instances Test, and Symbol Coding and Tower of London Test, respectively. Each patient’s performance in the individual tests is compared with a healthy comparison group to calculate the *T*- or *Z*-score of that sum (Keefe et al., 2008). We applied and computed statistical analysis using *T*-scores based on Taiwanese norms (Wang et al., 2017).

#### Affective processing test

The Affective Processing Tests are applied in addition to the six BAC-A subtests, and include the Affective Interference Test (AIT), Emotion Inhibition Test (EIT), and Delayed Recognition. A list of 20 words is given to the subjects in the Affective Interference Test, with half of the words featuring emotional content (affective words like “intimate” and “killer”) and the other half being fruits or vegetables (non-affective words like “zucchini” and “apple”). Three learning trials were given to the subjects, and each trial required them to recall as many of these terms as possible. The subjects were asked to freely recall the non-affective words (fruits and vegetables) and then the “other words”. We calculated the analyses using the following four indexes: (a) total non-affective words; (b) total affective words; (c) cued non-affective words, and (d) cued affective words.

Recognition memory is tested after a delay of 15–20 min by presenting the initial 20 words (10 fruits and vegetables and 10 emotional) with 20 foil words that had not already been presented. The subjects were asked if certain non-affective and affective words were previously included in the word list. Four indexes were assessed to analyze the AIT-Delayed Recognition, including (a) number of correct non-affective words; (b) number of correct affective words; (c) non-affective false alarms, and (d) affective false alarms.

Sheets of papers containing four columns of words of either affective polarity or neutral in colored (red, blue, green, and yellow) or black ink were presented to the subjects in the EIT. The subjects are then instructed to either read the color of the words (color naming) or the words (word naming) going down the columns. They are given 30 s to read as many words as they can. The Emotion Inhibition index is identified by subtracting: (a) the number of correct responses to the neutral color words; (b) the number of correct responses to the color naming; (c) the number of correct responses to neutral words, and (d) the number of correct responses to the affective color words;

### Psychopathological assessment

The diagnosis of depression was made/confirmed through face-to-face interview and chart review according to the criteria of DSM-IV-TR by psychiatrists. The clinical psychopathology of patients was evaluated using the 17-item Hamilton Depression Rating Scale (17-item HAM-D). The 17-item HAM-D consists of 17 items and is a clinician-rated scale for depressive symptoms (Ramos-Brieva & Cordero-Villafafila, 1988). A higher total score indicates a greater severity of depressive symptoms (Zheng et al., 1988). Furthermore, we recorded the duration of illness and psychotropic drugs in use (antipsychotics, antidepressants, mood stabilizers, and benzodiazepines) during the interview and by reviewing the patients’ medical records.

### Statistical analyses

The statistical software package SPSS (Version 21.0; SPSS Inc., Chicago, IL, USA) was used to analyze the data. The variables were presented as either frequency (%) or mean (±SD). We categorized the depressive patients into “those without residual symptoms” and “those with residual symptoms” based on a HAMD score of 7. Residual symptoms are classified by those symptoms that remained after treatment in either group of remission (defined as those patients who scores ≤7 in HAMD17) (Hiranyatheb et al., 2016). We used the chi-square test to compare the categorical variables among participant groups and the variables between depressive patients and controls, patients with depressive disorder NOS or dysthymic disorder (non-MDD group) and major depressive disorder (MDD group), as well as patients with and without residual symptoms. We performed group comparisons using *t*-tests for continuous variables, and Mann–Whitney *U* test was applied for group comparison when the continuous variables violated normal distribution. The gender- and age- adjusted *T*-scores were set as dependent variables in each BAC-A subtest.

Multivariate analysis of covariance (MANCOVA) was used for detecting the differences in BACA across groups (controlling for age, sex and education levels), with LSD post-hoc test. We also adopted multiple linear regression models to explore the effects of depression characteristics on the performance of each BAC-A subtest. The subtest-scores of the BAC-A were set as dependent variables, while we viewed depression characteristics (age, sex, education levels, HAMD-17 scores, duration of illness, antidepressant use and benzodiazepine use) as independent variables. Values were considered statistically significant if a two-tailed test <0.05. We used Bonferroni correction (0.05/19 = 0.0026) to adjust for multiple testing in BACA performance (a total of 19 indices).

## Results

The performance in the BAC-A and characteristics of the 75 patients with depression (mean age: 45.4 years, 36.0% males) and 75 age- and sex-matched controls (mean age: 46.1 years, 36.0% males) are shown in Table 1. The control group had a greater level of education than the patient group (*p* = 0.025). Regarding BAC-A performance, patients with depression had significantly worse performance in all indices, except executive function, cued affective words of the AIT, and four indexes of the AIT-Delayed Recognition.

We also compared the performance in the BAC-A and characteristics of the 56 patients in the MDD group (mean age: 46.6 years, 32.1% males) and 19 patients in the non-MDD group (mean age: 42.0 years, 47.4% males) (Table 2). After adjustment for education levels, no significant difference in any indexes of the BACA was found between the MDD group and non-MDD group.

**Table 1:** Characteristics and cognitive function of patients with depressive disorders and healthy controls.

	Depression (*n* = 75)	Healthy controls (*n* = 75)	Statistical value	*p*-value
Gender, *n* (%)			0.000	1.000
Male	27 (36.0)	27 (36.0)		
Female	48 (64.0)	48 (64.0)		
Age (years)	45.4 ± 12.1	46.1 ± 11.2	−0.337	0.737
Years of education	13.6 ± 3.1	14.7 ± 2.7	−2.270	0.025
Age of onset (years)	36.9 ± 12.3	–	–	–
Duration of illness (years)	8.5 ± 8.0	–	–	–
Mood episode (times)	1.8 ± 1.7	–	–	–
HAMD-17 items total scores	7.1 ± 4.4	–	–	–
Antidepressant use, *n* (%)	56 (74.7)	–	–	–
Duration of use (months)	28.3 ± 37.9	–	–	–
Defined daily dose	1.1 ± 0.7	–	–	–
Antipsychotics use, *n* (%)	21 (28.0)	–	–	–
Duration of use (months)	28.0 ± 34.0	–	–	–
Defined daily dose	0.3 ± 0.3	–	–	–
Benzodiazepine use, *n* (%)	60 (80.0)	–	–	–
Duration of use (months)	31.0 ± 35.1	–	–	–
Defined daily dose	1.3 ± 1.2	–	–	–
Cognitive assessment (BACA)				
BACS				
Verbal memory	38.7 ± 10.7	50.7 ± 10.2	38.957	<0.001^*^
Working memory	44.2 ± 9.7	50.4 ± 11.9	6.771	0.010
Motor speed	42.5 ± 10.3	51.0 ± 11.5	18.047	<0.001^*^
Verbal fluency	42.0 ± 10.7	52.1 ± 9.3	27.204	<0.001^*^
Attention and processing speed	37.8 ± 13.5	50.7 ± 10.8	31.663	<0.001^*^
Executive function	44.8 ± 15.8	48.9 ± 10.5	2.170	0.143
BACS Composite score	35.6 ± 15.8	50.7 ± 10.7	36.716	<0.001^*^
Affective Processing Test				
AIT: total affective words	41.6 ± 9.1	50.8 ± 10.4	24.865	<0.001^*^
AIT: total non-affective words	39.7 ± 13.4	50.3 ± 10.3	21.077	<0.001^*^
AIT: cued affective words	47.0 ± 10.1	50.6 ± 10.7	2.891	0.091
AIT: cued non-affective words	44.5 ± 9.8	50.4 ± 10.7	7.759	0.006
DR: correct affective words	51.5 ± 6.6	49.7 ± 10.7	2.256	0.135
DR: non-affective correct words	50.3 ± 6.2	49.8 ± 10.1	0.813	0.369
DR: affective false alarms	51.9 ± 9.5	50.7 ± 10.4	0.202	0.653
DR: non-affective false alarms	55.4 ± 13.8	50.3 ± 10.2	3.882	0.051
EIT: color naming score	45.0 ± 12.7	51.7 ± 10.0	7.224	0.008
EIT: neutral color word score	42.2 ± 13.8	51.0 ± 10.1	13.049	<0.001^*^
EIT: affective color word score	42.0 ± 12.4	51.0 ± 10.2	16.979	<0.001^*^
EIT: neutral word score	43.2 ± 12.5	49.7 ± 9.8	8.936	0.003

**Notes.**

Data are expressed as mean  ± SD or *n* (%). Bonferroni correction was used to adjust for multiple testing in the correlation matrix (*p*-value = 0.05/19 = 0.0026).

* Significant correlation remains after Bonferroni correction.

**Table 2:** Characteristics and cognitive function of patients with major depressive disorder (MDD group) and patients with dysthymic disorder or depressive disorder NOS (non-MDD group).

	MDD (*n* = 56)	Non-MDD (*n* = 19)	Statistical value	*p*-value
Gender, *n* (%)			1.427	0.232
Male	18 (32.1)	9 (47.4)		
Female	38 (67.9)	10 (52.6)		
Age (years)	46.6 ± 12.5	42.0 ± 10.0	1.780	0.075
Years of education	13.0 ± 3.0	15.3 ± 2.8	2.668	0.008
Age of onset (years)	37.7 ± 12.4	34.5 ± 11.9	1.042	0.297
Duration of illness (years)	8.9 ± 7.8	7.5 ± 8.8	1.143	0.253
Mood episode (times)	2.0 ± 1.9	1.2 ± 0.9	2.608	0.009
HAMD-17 items total scores	7.4 ± 4.5	6.1 ± 4.0	0.984	0.325
Antidepressant use, *n* (%)	42 (75.0)	14 (73.7)	0.013	0.909
Duration of use (months)	30.5 ± 36.0	21.6 ± 44.0	1.629	0.103
Defined daily dose	1.2 ± 0.7	0.9 ± 0.5	1.816	0.069
Antipsychotics use, *n* (%)	18 (32.1)	3 (15.8)	1.882	0.170
Duration of use (months)	32.17 ± 35.0	2.7 ± 2.9	2.514	0.006
Defined daily dose	0.3 ± 0.4	0.1 ± 0.1	0.682	0.523
Benzodiazepine use, *n* (%)	47 (83.9)	13 (68.4)	2.132	0.144
Duration of use (months)	36.8 ± 37.5	9.9 ± 13.5	3.072	0.002
Defined daily dose	1.4 ± 1.3	0.9 ± 0.5	1.368	0.171
Cognitive assessment (BACA)				
BACS				
Verbal memory	37.8 ± 11.7	41.1 ± 7.2	0.037	0.848
Working memory	43.4 ± 9.3	46.6 ± 10.7	0.055	0.815
Motor speed	41.7 ± 11.2	44.9 ± 7.2	0.007	0.935
Verbal fluency	40.3 ± 10.3	46.8 ± 10.7	0.836	0.364
Attention and processing speed	35.8 ± 13.7	43.2 ± 11.5	1.250	0.268
Executive function	43.3 ± 17.5	48.8 ± 9.0	0.853	0.359
BACS Composite score	33.4 ± 16.9	41.7 ± 10.5	1.074	0.304
Affective Processing Test				
AIT: total affective words	42.0 ± 9.9	40.6 ± 6.7	2.620	0.110
AIT: total non-affective words	37.4 ± 13.1	46.3 ± 12.2	2.069	0.155
AIT: cued affective words	46.7 ± 11.2	47.5 ± 6.2	0.467	0.497
AIT: cued non-affective words	43.5 ± 10.4	47.2 ± 7.5	0.029	0.866
DR: correct affective words	50.9 ± 7.2	53.4 ± 4.3	0.349	0.557
DR: non-affective correct words	49.2 ± 6.7	53.3 ± 3.1	2.351	0.130
DR: affective false alarms	52.7 ± 10.1	49.6 ± 7.3	0.402	0.528
DR: non-affective false alarms	57.1 ± 14.7	50.6 ± 9.5	1.140	0.290
EIT: color naming score	43.5 ± 12.7	49.1 ± 11.9	0.264	0.609
EIT: neutral color word score	40.9 ± 14.4	45.6 ± 11.6	0.006	0.939
EIT: affective color word score	41.3 ± 12.9	44.1 ± 11.0	0.048	0.827
EIT: neutral word score	43.3 ± 13.1	42.7 ± 11.0	0.793	0.376

**Notes.**

Data are expressed as mean  ± SD or *n* (%).

MDD major depressive disorder

Bonferroni correction was used to adjust for multiple testing in the correlation matrix (*p*-value = 0.05/19 = 0.0026).

We further compared the performance in the BAC-A of the 31 patients with residual symptoms (mean age: 46.1 years, 41.9% males), 44 patients without residual symptoms (mean age: 45.0 years, 31.8% males) and healthy controls (Table 3). We found that patients with residual symptoms had worst performance in working memory, motor speed, verbal fluency, attention, executive function, composite BACS score, three indexes of the AIT and all four indexes of the EIT. Additionally, patients without residual symptoms exhibited intermediate performance (between those with residual symptoms and healthy controls) in motor speed, verbal fluency, attention, composite BACS score, total non-affective words of the AIT and affective color word scores of the EIT.

**Table 3:** Characteristics and cognitive function of depression patients with residual symptoms, patients without residual symptoms and healthy controls.

	Residual (A) (*n* = 31)	No-residual (B) (*n* = 44)	Healthy controls (C) (*n* = 75)	*χ*^2^ or *F*	*p*-value	*Post-hoc test*
Gender, *n* (%)				0.808	0.668	
Male	13 (41.9)	14 (31.8)	27 (36.0)			
Female	18 (58.1)	30 (68.2)	48 (64.0)			
Age (years)	46.1 ± 12.4	45.0 ± 12.0	46.1 ± 11.2	0.136	0.873	
Years of education	13.0 ± 3.3	14.1 ± 2.9	14.7 ± 2.7	3.860	0.023	C >A
Cognitive assessment (BACA)						
BACS						
Verbal memory	36.6 ± 10.3	40.1 ± 10.9	50.7 ± 10.2	20.059	<0.001^*^	C >A, C >B
Working memory	39.8 ± 9.6	47.1 ± 8.8	50.4 ± 11.9	6.418	0.002^*^	C >A, B >A
Motor speed	37.9 ± 9.7	45.6 ± 9.7	51.0 ± 11.5	13.335	<0.001^*^	C >B >A
Verbal fluency	37.9 ± 12.4	44.7 ± 8.6	52.1 ± 9.3	16.907	<0.001^*^	C >B >A
Attention and processing speed	32.2 ± 11.8	41.5 ± 13.4	50.7 ± 10.8	20.656	<0.001^*^	C >B >A
Executive function	40.1 ± 21.6	47.9 ± 9.5	48.9 ± 10.5	3.614	0.030	C >A, B >A
BACS Composite score	28.2 ± 17.7	40.4 ± 12.4	50.7 ± 10.7	26.133	<0.001^*^	C >B >A
Affective Processing Test						
AIT: total affective words	40.1 ± 8.7	42.6 ± 9.4	50.8 ± 10.4	12.702	<0.001^*^	C >A, C >B
AIT: total non-affective words	34.2 ± 11.1	43.4 ± 13.6	50.3 ± 10.3	15.229	<0.001^*^	C >B >A
AIT: cued affective words	42.6 ± 8.9	49.8 ± 10.0	50.6 ± 10.7	5.018	0.008	C >A, B >A
AIT: cued non-affective words	40.2 ± 9.6	47.3 ± 9.0	50.4 ± 10.7	7.360	0.001^*^	C >A, B >A
DR: correct affective words	48.9 ± 8.1	53.3 ± 4.8	49.7 ± 10.7	2.931	0.057	–
DR: non-affective correct words	49.6 ± 5.8	50.7 ± 6.4	49.8 ± 10.1	.430	0.652	–
DR: affective false alarms	54.7 ± 10.2	50.0 ± 8.7	50.7 ± 10.4	1.736	0.180	–
DR: non-affective false alarms	55.4 ± 11.0	55.3 ± 15.5	50.3 ± 10.2	1.970	0.143	–
EIT: color naming score	39.6 ± 10.6	48.6 ± 12.8	51.7 ± 10.0	8.208	<0.001^*^	C >A, B >A
EIT: neutral color word score	36.5 ± 10.9	45.9 ± 14.3	51.0 ± 10.1	11.164	<0.001^*^	C >A, B >A
EIT: affective color word score	37.0 ± 9.4	45.3 ± 13.2	51.0 ± 10.2	12.746	<0.001^*^	C >B >A
EIT: neutral word score	38.9 ± 10.3	46.0 ± 13.2	49.7 ± 9.8	7.483	0.001^*^	C >A, B >A

**Notes.**

Data are expressed as mean  ± SD or *n* (%). Statistical Value are estimated using MANCOVA (controlling for age, sex and education levels).

Residual the HAMD scores >7 No-residual HAMD scores ≤7 A patients with residual symptoms B patients without residual symptoms C healthy controls

Bonferroni correction was used to adjust for multiple testing in the correlation matrix (*p*-value = 0.05/19 = 0.0026).

* Significant correlation remains after Bonferroni correction.

We further used multiple linear regression to explore the effects of depression characteristics on the performance of each BAC-A index (Table 4). After controlling for patients’ age, sex and educational levels, we found that the past MDD diagnoses were not significantly associated with worse cognitive function than non-MDD patients. The HAMD-17 scores were negatively correlated to working memory, motor speed, verbal fluency, attention and processing speed, executive function, the BACS composite score, cued affective words and cued non-affective words of the AIT, and all four indexes of the EIT. Furthermore, benzodiazepine use was associated with worse performance of verbal memory; antidepressant use was correlated with better performance of motor speed; and a longer illness duration was associated with worse performance of total non-affective words of the AIT and three indexes of the EIT.

**Table 4:** Effects of illness characteristics and psychotropic drugs on the performance of each index of the BACA.

BACA index	Associated factors	B (95% CI)	Std. error	*t*	*p*-value	Observed power
Verbal memory	Benzodiazepine use	−9.82 (−17.00, −2.63)	3.589	−2.735	0.008	0.767
Working memory	HAMD-17 scores	−0.77 (−1.38, −0.17)	0.303	−2.554	0.013	0.709
Motor speed	HAMD-17 scores	−1.24 (−1.89, −0.59)	0.326	−3.804	<0.001	0.963
	Antidepressant use	8.02 (2.01, 14.04)	3.004	2.670	0.010	0.747
Verbal fluency	HAMD-17 scores	−0.82 (−1.47, −0.16)	0.327	−2.495	0.015	0.689
Attention and processing speed	HAMD-17 scores	−1.24 (−2.10, −0.38)	0.429	−2.887	0.005	0.810
Executive function	HAMD-17 scores	−1.19 (−2.31, −0.07)	0.560	−2.119	0.038	0.549
BACS Composite score	HAMD-17 scores	−1.86 (−2.83, −0.89)	0.484	−3.842	<0.001	0.965
AIT: total affective words	NS					
AIT: total non-affective words	Duration of illness	−0.53 (−0.92, −0.15)	0.193	−2.762	0.008	0.775
AIT: cued affective words	HAMD-17 scores	−0.83 (−1.49, −0.18)	0.325	−2.565	0.013	0.713
AIT: cued non-affective words	HAMD-17 scores	−0.83 (−1.41, −0.26)	0.287	−2.908	0.005	0.816
DR: correct affective words	NS					
DR: non-affective correct words	NS					
DR: affective false alarms	NS					
DR: non-affective false alarms	NS					
EIT: color naming score	HAMD-17 scores	−1.16 (−1.92, −0.39)	0.381	−3.035	0.004	0.847
	Duration of illness	−0.45 (−0.84, −0.06)	0.195	−2.328	0.023	0.629
EIT: neutral color word score	HAMD-17 scores	−1.34 (−2.17, −0.52)	0.413	−3.250	0.002	0.892
EIT: affective color word score	Duration of illness	−0.37 (−0.73, −0.01)	0.181	−2.045	0.045	0.520
	HAMD-17 scores	−1.13 (−1.90, −0.36)	0.383	−2.946	0.005	0.826
EIT: neutral word score	Duration of illness	−0.51 (−0.90, −0.12)	0.194	−2.645	0.011	0.739
	HAMD-17 scores	−1.36 (−2.18, −0.54)	0.410	−3.323	0.002	0.904

**Notes.**

All models were controlled for age, sex and education levels. Only the factors with significant effect were shown in the Table.

AIT Affective Interference Test DR Affective Interference Test: Delayed Recognition EIT the Emotion Inhibition Test MDD major depressive disorder NS none of the factors showed significant effect

## Discussion

**Figure 1:**
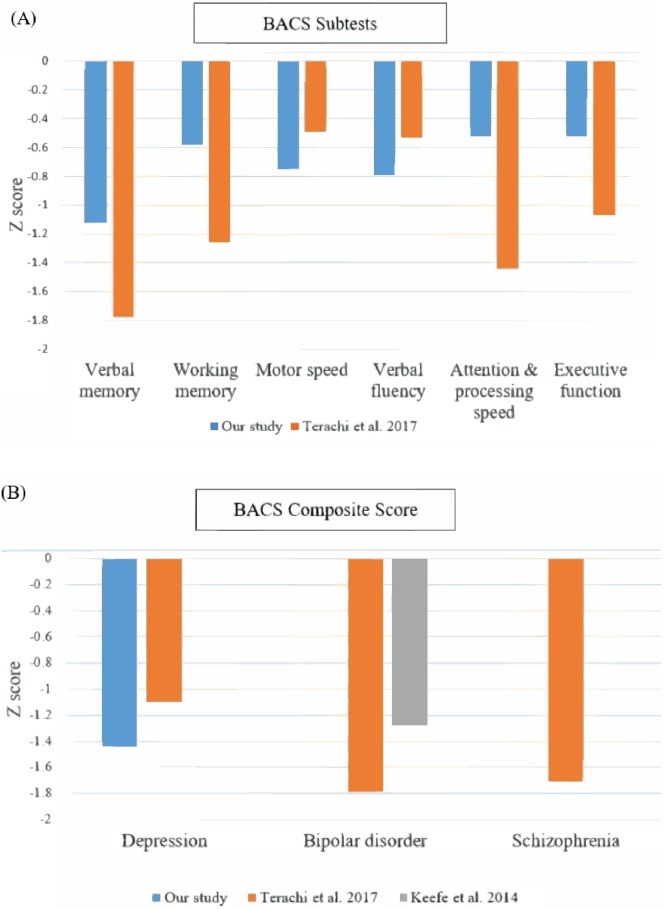
Comparisons of cognitive performance of our patients with depressive disorders with previous studies using BACS as an assessment tool. (A) Comparison of performance of each BACS subtest in patients with depressive disorder (Compared to Terachi et al. (2017)). (B) Comparison of BACS composite score in patients with depressive disorder, bipolar disorder and schizophrenia (Compared to Terachi et al. (2017) and Keefe et al. (2014)).

In our study, depressive patients showed impairments in many domains of BACA, including verbal memory, working memory, motor speed, verbal fluency, attention and speed of information processing. The magnitude of cognitive dysfunction was compatible to which reported in a previous study (using BACS to assess cognitive function among MDD patients, Fig. 1A) (Terachi et al., 2017). We also compared our results with previous studies with bipolar disorder and schizophrenia patients conducting BACS tests (Fig. 1B). Similar degrees of cognitive impairments were observed in patients with bipolar disorder and schizophrenia patients, while patients with MDD may exhibited smaller impairments in several neurocognitive domains compared to patients with either of the other two disorders (Terachi et al., 2017).

In this study, we aimed to define the cognitive profiles of patients with depressive disorders using the BAC-A and found that HAMD-17 scores were positively correlated with cognitive dysfunction in motor speed, verbal fluency, attention and processing speed, and executive function domains measured using the BAC-A. A previous study suggested that cognitive dysfunction may remain in the remitted state of depression, which gives rise to whether the severity of depression is the only component affecting cognitive function. In line with our findings, another study indicated that cognitive performance was significantly associated with depression severity. Notably, we found no differences in cognitive profiles in the MDD group and non-MDD group after adjusting for potential confounding factors. This result implies that the current residual depressive symptom severity, not the diagnosis categories, was most closely related to cognitive deficits among patients with depressive disorders.

Symptoms of depressive disorder, such as lack of interest, impaired processing initiative, lack of motivation, and attention deficit, impact the performance of memory function (Marazziti et al., 2010; Ellis, 1990) and are closely related to the depression course regarding current mood states (Bartfai et al., 1991; MacQueen et al., 2002). In a previous study, perceived cognitive performance and residual depressive symptoms were found to be significantly correlated to psychosocial functioning in patients with affective disorder (Samalin et al., 2017). Our studies further identified that residual depressive symptoms was a significant indicator for cognitive impairments in many domains of the BACA. Affective performance biases such as problems with pessimistic attributional style, automatic thoughts, levels of neuroticism and attentional control problems in MDD patients during remission had been discussed before (Cerny et al., 2019). As for delayed recognition, it is worth noting that unipolar depression patients committed more non-affective false alarms during delayed recognition compared to patients with bipolar disorder and healthy controls (Lee et al., 2018b). This result may be due to the fact that depressed patients tend to process negative information preferably  (Siegle & Hasselmo, 2002).

With regard to the relationship between psychotropic drugs and cognitive function, we found that benzodiazepine use was associated with worse verbal memory performance. Previous studies have offered support indicating consistent impairment in multiple cognitive domains, including verbal memory, in long-term benzodiazepine users regarding psychiatric illness and in people under withdrawal and those who had abstained. However, we did not discuss the duration of benzodiazepine usage or withdrawal status in this study. Although the exact mechanism of how benzodiazepine affects cognitive function has remained unclear, the alternation of the *α*1-GABAA receptor subtype by benzodiazepine may play a role in cognitive function performance (Makaron et al., 2013).

We found that antidepressant use was associated with better performance of motor speed. In line with our study’s finding, a recent meta-analysis also revealed a positive effect of antidepressants on delayed recall and psychomotor speed (Rosenblat, Kakar & McIntyre, 2015). One previous study suggested that verbal learning and memory were particularly impaired in patients with depression and were sensitive to the effects of certain selective serotonin reuptake inhibitors (SSRIs) (Schmitt, Kruizinga & Riedel, 2001). As Schmitt reported, delayed recall in word learning had been significantly impaired by paroxetine at the doses of 20 mg and 40 mg, while sertraline has been reported to impact word learning but improve verbal fluency. The enhancement of monoamines (dopamine, norepinephrine and serotonin) by antidepressants may be related to better cognitive function (Trivedi et al., 2008). However, our studies demonstrated that neither antidepressant nor antipsychotics usage was correlated with verbal memory or working memory function in depressed patients. A randomized longitudinal study recently demonstrated that no significant improvement was shown in several cognitive domains in depressed patients following medication treatment (Shilyansky et al., 2016). Therefore, a longitudinal follow-up is needed to clarify whether antidepressant treatment actually improves the performance of motor speed among depressive patients.

We have found that duration of illness is significantly correlated to total non-affective words and three subtest of the Emotion Inhibition Test. Which indicate that valence of past period might play roles by which depressed patients might have difficulty processing positive stimuli (Gotlib et al., 2011). Verbal memory is found to be correlated to benzodiazepine use and education level in our study. The result is similar to previous research conducted by Bas and Poyraz (Bas et al., 2015) that indicate low functioning bipolar patients perform significantly worse in verbal memory in terms that low functioning patients tends to receive lower level of education and greater amount of medication for their symptoms. However, the high risk of developing major depressive disorder in low education group had been identified in some previous studies indicating that environmental or genetic factors may play roles (Lorant et al., 2003; Peyrot et al., 2015) which is compatible to our data indicating non-MDD group tends to receive longer period of education.

This study has several limitations that should be noted. First, the sample size of this study was small, particularly the non-MDD group. Therefore, negative findings in MDD-related cognitive decline may be due to the small sample size. Besides, homogeneity of variance between the MDD and non-MDD groups was lack due to limited sample size. Second, the age and gender were not perfectly matched between the MDD group and the non-MDD group, and the case numbers were unequal. Differences in educational level and psychotropic drugs in use across groups also played confounding factors in the investigation for cognitive performance. Third, several crucial factors potentially associated with cognitive function (e.g., premorbid function, smoking, alcohol use, comorbidities and treatment response to medication) were not addressed in this study. Additionally, we did not collected the information about socioeconomic status, average/quality of sleep prior to testing, use of coffee or nicotine during the day of testing and immediately prior to testing. Future studies with larger sample sizes and comprehensive psychopathological assessments are necessary to understand the influence that these aforementioned factors may have on between-group neurocognitive differences.

## Conclusions

In conclusion, we found that the current severity of depressive symptoms and psychotropic in use, not the diagnosis category, were associated with cognitive impairments in patients with depressive disorders. Clinicians should make sure to manage residual depressive symptoms and prescribe adequate psychotropic drugs in order to eliminate the cognitive deficits of depressive patients. BACA is a reliable instrument to detect cognitive deficits in depressive patients even with minimal residual symptoms. However, future studies with adequate statistic power and comprehensive psychopathological assessments are necessary to understand the neurocognition-related factors among depressive patients.

##  Supplemental Information

10.7717/peerj.7432/supp-1 Table S1 Psychotropic drugs in use and mean daily dose among patients with depressive disorders

10.7717/peerj.7432/supp-2 Dataset S1 Raw data of BACA performance in patients with depressive disorders

## Additional Information and Declarations

### Data Availability

The following information was supplied regarding data availability: The raw measurements are available as Supplemental File.
