# Supplementary material for: Defining cognitive profiles of depressive patients using the Brief Assessment of Cognition in Affective Disorders
Source: PeerJ. 2019 Aug 1;7:e7432. doi: 10.7717/peerj.7432 (PMC6679906; doi:10.7717/peerj.7432)
Supplement: Table S1 [file peerj-07-7432-s001.docx]

**Supplementary Table 1** Psychotropic drugs in use and mean daily dose among patients with depressive disorders

| **Categories** | **Drugs** | **Case number** | **Mean Daily Dose** |
| --- | --- | --- | --- |
| Antidepressants | Fluoxetine | 2 | 20mg |
|  | Paroxetine | 5 | 20mg |
|  | Escitalopram | 13 | 10mg |
|  | Bupropion | 5 | 150mg |
|  | Venlafaxine | 6 | 56.3mg |
|  | Duloxetine | 8 | 37.5mg |
|  | Mirtazapine | 7 | 30mg |
|  | Agomelatine | 9 | 25mg |
|  | Trazodone | 6 | 50mg |
|  |  |  |  |
| Antipsychotics | Risperidone | 2 | 1.5mg |
|  | Quetiapine | 13 | 25mg |
|  | Aripiprazole | 4 | 7.5mg |
|  | Sulpiride | 2 | 75mg |
|  |  |  |  |
| Benzodiazepines | Lorazepam | 17 | 1.1mg |
|  | Alprazolam | 22 | 0.5mg |
|  | Diazepam | 2 | 2mg |
|  | Clonazepam | 19 | 1.6mg |
|  |  |  |  |
| Others | Diphenhydramine | 1 | 50mg |
|  | Buspirone | 2 | 10mg |
|  | Amitriptyline | 2 | 25mg |
|  | Valproate sodium | 4 | 425mg |
